# What lies beyond 100 years of insulin

**DOI:** 10.1242/dmm.049361

**Published:** 2021-11-09

**Authors:** Kirsty M. Hooper

**Affiliations:** The Company of Biologists, Bidder Building, Station Road, Cambridge CB24 9LF, UK

**Keywords:** Diabetes, Insulin, Metabolism

## Abstract

It has been 100 years since the discovery of insulin. This revolutionary treatment saves the lives of millions of people living with diabetes, but much remains to be understood of its mechanisms and roles in homeostasis and disease. To celebrate this centenary, we explore areas of ongoing insulin research in diabetes, metabolic syndrome and beyond. Disease Models & Mechanisms aims to publish high-quality basic and pre-clinical research that advances our understanding of these conditions to facilitate clinical and public health impact.

There was a time when diagnosis with type 1 diabetes (T1D) was a death sentence. Patients would merely have days or weeks and, if lucky, months to live. Life expectancy could be extended marginally with drastic ketogenic diets that restricted carbohydrate intake; but this led to severe malnutrition and, in the best-case scenario, survival would be extended by one to two years. In 1921, surgeon Frederick Banting, returning from World War I, worked with student Charles Best and had a breakthrough in treating canine diabetes with pancreatic extracts of insulin at John Macleod's laboratory in the University of Toronto. Banting, Best and Macleod then collaborated with the biochemist James Collip to further purify the insulin extracted from cows and proceeded to treat their first patient, 14-year-old Leonard Thompson. For developing this miracle treatment, the scientists won the 1923 Nobel Prize, and global production of insulin was rapidly expanded to treat as many patients as possible.

Over the next 100 years, insulin therapy and diabetes management have continued to improve. Insulin with extended action was developed, a Nobel Prize was procured by Frederick Sanger in 1958 for mapping the structure of the hormone, and genetically engineered human insulin was developed in 1978 and commercialised in 1982. In 1959, another key moment occurred with the classification of insulin-dependent (type 1) and insulin-independent (type 2) diabetes. Fast forward to 2019, when 463 million adults worldwide were suffering from type 1 or type 2 diabetes (T1D or T2D, respectively), a figure that had tripled over the past 20 years, with ~90% of cases being T2D (International Diabetes Federation, 2019). Further research is desperately required to tackle the T2D pandemic and to improve the treatment of T1D, and the landmark discoveries made over the past 100 years have paved the way for this.

**Figure:**
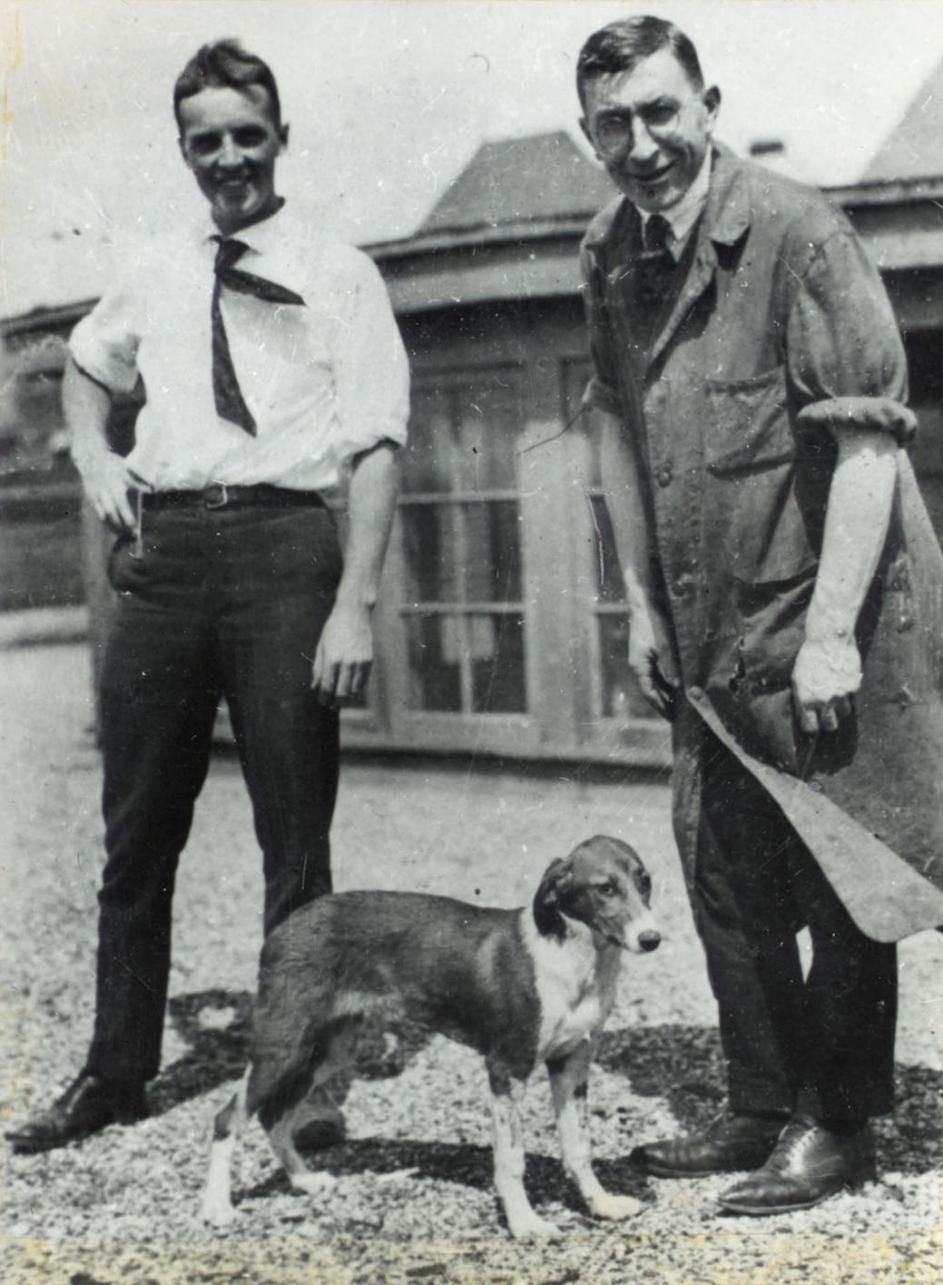
**Frederick Banting and Charles Best with a dog on the roof of the Medical Building, University of Toronto, 1921.** This image was originally posted to Flickr by the Thomas Fisher Rare Book library at https://www.flickr.com/photos/34861056@N05/12309019434. It is reproduced without modification under the terms of the Creative Commons Attribution 2.0 Generic license.

Disease Models & Mechanisms (DMM) articles represent stellar examples of the variety of research taking place to advance our understanding of the mechanisms of diabetes and other conditions related to insulin dysregulation. We have compiled a collection dedicated to Metabolic Disorders. In this issue, we celebrate the centenary of insulin's discovery with ‘A Model for Life’ interview with Dr Elizabeth Seaquist (Sequist, 2021), and Perspective articles from Dr Eva Feldman (Eid and Feldman, 2021) and Dr Robert Semple (Brierley and Semple, 2021). By promoting this research, we aim to support the improved management and treatment of insulin-related conditions, many of which are rapidly growing globally with devastating consequences.

T1D is characterised by the loss of insulin-producing β-cells in the pancreas due to a cytokine-mediated autoimmune reaction. Type 2 diabetes (T2D) is associated with overnutrition and a lack of physical activity. A prominent feature of early T2D – or prediabetes – is insulin resistance, the systemic lack of responsiveness to insulin discussed in detail in the Perspective by Brierley and Semple (Brierley and Semple, 2021). This prediabetic state can also be exacerbated in pregnancy, leading to gestational diabetes that, in a pig model, was shown to cause impaired glucose tolerance, insulin resistance and differential metabolomic profiles in the neonatal piglets (Renner et al., 2019). This highlights the potential risk of T2D in the children of mothers with gestational diabetes.

Prediabetes progresses towards T2D through inflammation of pancreatic islets, leading to β-cell damage and dysfunction, and resulting in deficient insulin production. This inflammation is associated with overexpression of the pro-inflammatory cytokines interferon gamma (IFNγ), interleukin 1 beta (IL1β) and tumour necrosis factor alpha (TNFα). In zebrafish, pancreatic overexpression of IL1β alone (Delgadillo-Silva et al., 2019) or of all three cytokines (Ibrahim et al., 2020) increases macrophage recruitment to the islets and NFκB signalling, as well as levels of reactive oxidative species and endoplasmic reticulum stress in β-cells, leading to their dysfunction and death. In zebrafish that overexpress all three cytokines, the medicinal plant derivative wedelolactone reduces islet macrophage infiltration and the hyperglycaemic phenotype (Delgadillo-Silva et al., 2019). These studies not only exhibit the role of islet inflammation in diabetes pathogenesis but also highlight the contribution of zebrafish to forward diabetes research. Importantly, many current treatments are aimed at the management of diabetes symptoms, but understanding islet inflammation could help develop therapies that prevent the progression of T2D.

The liver has a fundamental role in insulin-related conditions because insulin resistance and/or deficiency diminishes the control of glucose secretion from the liver, which – together with impaired glucose uptake by systemic cells and tissues – leads to hyperglycaemia. Insulin also inhibits the breakdown of triglycerides in adipose tissue, meaning its impaired action causes accumulation of fatty acids and lipids in the liver. Pathologies of the liver, therefore, often occur together with insulin resistance and/or T2D, and many encompass metabolic syndrome, a collection of metabolism-related dysfunctions that are often associated with obesity and increase the risk of T2D. One example is hepatic steatosis, also known as fatty liver, which is characterised by intrahepatic fat accumulation; another is dyslipidaemia, which manifests itself in increased levels of lipids in the blood. Diet-induced animal models of insulin resistance and obesity, including mouse (Lopez-Pastor et al., 2019; Villar-Lorenzo et al., 2019), rat (Bacle et al., 2020) and sheep (Kalyesubula et al., 2021) models, recapitulate many of the liver-associated conditions.

Mouse models of liver pathologies with a stronger genetic basis – such as congenital generalised lipodystrophy, a condition that can lead to diabetes (Mcilroy et al., 2020), or glycogen storage disease type 1a (GSD1A) that is associated with hepatocellular tumours (Resaz et al., 2020) – have not been as clearly linked to insulin resistance; there is, however, evidence of hyperinsulinemia and altered insulin signalling, regarding congenital generalised lipodystrophy (Mcilroy et al., 2020) and GSD1A (Resaz et al., 2020), respectively. These studies highlight the breadth of impact insulin has on glucose and lipid metabolism, and on the potential resultant liver pathologies.

As there is currently no cure for diabetes, this life-long condition manifests itself in complications affecting organs beyond the pancreas and liver, such as the kidneys, and the peripheral and central nervous systems. Diabetic kidney disease (DKD) accounts for 25–50% of patients with kidney failure in Europe and the USA (Sembach et al., 2021). Advances in transcriptomic profiling have enabled deeper investigation of the molecular signalling pathways involved in development and progression of DKD in rodent models (Otto et al., 2019; Sembach et al., 2021), which is essential to identify novel targets for preventing or halting DKD. Peripheral neuropathy (PN) is a complication that occurs in 30% of prediabetes and 60% of T2D patients, and can lead to chronic pain and lower-limb amputations. Eva Feldman’s lab has vastly advanced research regarding PN, for example, in studies mapping the role of dyslipidaemia and altered lipid signalling (O'Brien et al., 2020) or identifying sex differences in its development (Elzinga et al., 2021). Eid and Feldman's Perspective article further explores the use of rodent PN models to enhance our understanding of this debilitating condition (Eid and Feldman, 2021). As metabolism is integral to neural cell development and function, alterations in the brain have also been linked to diabetes, as discussed in our interview with Dr Elizabeth Seaquist (Sequist, 2021). Novel laboratory model systems have been developed to investigate this phenomenon, including cultured neurons derived from T2D mice (De Gregorio and Ezquer, 2021) and zebrafish embryos that, when incubated in glucose, exhibit dysfunction in the neurovascular system and present behavioural alterations (Chhabria et al., 2019). These systems are attractive tools for identifying novel therapies for diabetes-associated complications of the central nervous system. Overall, these diabetes-associated complications emphasize the necessity for a new approach to treating diabetes, i.e. one that aims to cease or potentially reverse its progression.

Often overlooked is the role of aberrant insulin signalling in pathologies other than diabetes. Lipotoxicity, which is known to mediate insulin resistance, was most likely caused by insulin resistance and oxidative stress in the kidneys of a renal fibrosis mouse model (Escasany et al., 2021). Lipotoxicity can also cause cardiac steatosis in a condition known as lipotoxic cardiomyopathy (Walls et al., 2020). Cardiac function was restored in a *Drosophila* model of lipotoxic cardiomyopathy by altering the lipid metabolism to improve insulin sensitivity (Walls et al., 2020). There has also been concentrated focus on the roles metabolism and insulin signalling have in cancer, as diabetes and obesity are prominent risk factors for cancer. In their recent review, Lam Wong and Verheyen (2021) explain how tumour survival, growth and metastasis are promoted by dysregulated insulin signalling and insulin receptor expression in *Drosophila* models of T2D. It was suggested that components of the insulin signalling pathway present in the circulation act as biomarkers for certain cancers. Furthermore, metabolic alterations have been associated with neurodevelopmental and psychiatric disorders, as reviewed by Traxler et al. (2021), and insulin resistance has been implicated in polycystic ovary syndrome, as discussed in the Perspective by Brierley and Semple, (2021). By appreciating the biological connections between all insulin-related conditions, we can expand our understanding of the integral role insulin has in the body.

However, there is much to be achieved in the future of insulin research. Novel forms of insulin continue to be developed, such as oral, glucose-sensitive and liver-specific insulin, and new devices, such as automated insulin delivery systems, have the potential to transform diabetes management. There are also promising strategies to, possibly, cure diabetes. Several studies have demonstrated that it is possible to regenerate new β-cells through proliferation of existing β-cells or trans-differentiation of other cell types, raising the hope that diabetes can be cured through restoration of functional β-cell mass (Sims et al., 2021). In 2000, the ‘Edmonton Protocol’ was published describing islet transplantation in T1D (Shapiro et al., 2000), and recent advances in stem cell technology – with clinical trials ongoing – have refined this strategy (Sims et al., 2021). However, many hurdles remain, including immunoreactivity towards implanted islets and the potential development of malignancies. Although these approaches seem propitious, extensive research and exploration is required before these treatments can confidently be deemed safe and effective. One study that aims to improve the assessment of β-cell regeneration therapies, has tracked β-cell proliferation in the islets of mice by using newly enhanced 3D quantitative imaging (Roostalu et al., 2020). In terms of prevention of T2D, many opportunities are available but, despite this, its prevalence is rapidly growing in adults and, worryingly, also in children. Obesity is a major driving force of T2D. Further research is, therefore, required to understand this association and to formulate effective interventions. This research should be coupled with advocacy and government public health policies. By supporting foundational and pre-clinical research regarding diabetes and other insulin-related conditions, DMM provides a platform for this research and encourages its translation to both the clinic and public health policies.

## References

[DMM049361C1] Bacle, A., Kadri, L., Khoury, S., Ferru-Clément, R., Faivre, J.-F., Cognard, C., Bescond, J., Krzesiak, A., Contzler, H., Delpech, N. et al. (2020). A comprehensive study of phospholipid fatty acid rearrangements in metabolic syndrome: correlations with organ dysfunction. *Dis. Model Mech.* 13, dmm043927. 10.1242/dmm.043927 32303571 PMC7328154

[DMM049361C2] Brierley, G. V. and Semple, R. K. (2021). Insulin at 100 years: toxic mediator of obesity-related disease?. *Dis. Model Mech.* 14, dmm 049340. 10.1242/dmm.049340 10.1242/dmm.049340 PMC8649170 34841432

[DMM049361C3] Chhabria, K., Vouros, A., Gray, C., MacDonald, R. B., Jiang, Z., Wilkinson, R. N., Plant, K., Vasilaki, E., Howarth, C. and Chico, T. J. A. (2019). Sodium nitroprusside prevents the detrimental effects of glucose on the neurovascular unit and behaviour in zebrafish. *Dis. Model Mech.* 12. dmm039867. 10.1242/dmm.039867 31481433 PMC6765192

[DMM049361C4] De Gregorio, C. and Ezquer, F. (2021). Sensory neuron cultures derived from adult db/db mice as a simplified model to study type-2 diabetes-associated axonal regeneration defects. *Dis. Model Mech.* 14, dmm046334. 10.1242/dmm.046334 33262160 PMC7847260

[DMM049361C5] Delgadillo-Silva, L. F., Tsakmaki, A., Akhtar, N., Franklin, Z. J., Konantz, J., Bewick, G. A. and Ninov, N. (2019). Modelling pancreatic β-cell inflammation in zebrafish identifies the natural product wedelolactone for human islet protection. *Dis. Model Mech.* 12, dmm036004. 10.1242/dmm.036004 30679186 PMC6361155

[DMM049361C6] Eid, S. A. and Feldman, E. L. (2021). Advances in diet-induced rodent models of metabolically acquired peripheral neuropathy. *Dis. Model Mech.* 14, dmm 049337. 10.1242/dmm.049337 PMC8592018 34762126

[DMM049361C7] Elzinga, S. E., Savelieff, M. G., O'Brien, P. D., Mendelson, F. E., Hayes, J. M. and Feldman, E. L. (2021). Sex differences in insulin resistance, but not peripheral neuropathy, in a diet-induced prediabetes mouse model. *Dis. Model Mech.* 14, dmm048909. 10.1242/dmm.048909 33692086 PMC8077554

[DMM049361C8] Escasany, E., Lanzón, B., García-Carrasco, A., Izquierdo-Lahuerta, A., Torres, L., Corrales, P., Rodríguez Rodríguez, A. E., Luis-Lima, S., Martínez Álvarez, C., Javier Ruperez, F. et al. (2021). Transforming growth factor β3 deficiency promotes defective lipid metabolism and fibrosis in murine kidney. *Dis. Model Mech.* 14, dmm048249. 10.1242/dmm.048249 34431499 PMC8489029

[DMM049361C9] Ibrahim, S., Harris-Kawano, A., Haider, I., Mirmira, R. G., Sims, E. K. and Anderson, R. M. (2020). A novel Cre-enabled tetracycline-inducible transgenic system for tissue-specific cytokine expression in the zebrafish: CETI-PIC3. *Dis. Model Mech.* 13, dmm042556. 10.1242/dmm.042556 32457041 PMC7328138

[DMM049361C10] *International Diabetes Federation*, IDF Diabetes Atlas, 9th edn., Brussels, Belgium 2019.

[DMM049361C11] Kalyesubula, M., Mopuri, R., Asiku, J., Rosov, A., Yosefi, S., Edery, N., Bocobza, S., Moallem, U. and Dvir, H. (2021). High-dose vitamin B1 therapy prevents the development of experimental fatty liver driven by overnutrition. *Dis. Model Mech.* 14, dmm048355. 10.1242/dmm.048355 33608323 PMC7988776

[DMM049361C12] Lam Wong, K. K. and Verheyen, E. M. (2021). Metabolic reprogramming in cancer: mechanistic insights from *Drosophila*. *Dis. Model Mech.* 14, 1-17. 10.1242/dmm.048934 34240146 PMC8277969

[DMM049361C13] Lopez-Pastor, A. R., Gomez-Hernandez, A., Diaz-Castroverde, S., Gonzalez-Aseguinolaza, G., Gonzalez-Rodriguez, A., Garcia, G., Fernandez, S., Escribano, O. and Benito, M. (2019). Liver-specific insulin receptor isoform A expression enhances hepatic glucose uptake and ameliorates liver steatosis in a mouse model of diet-induced obesity. *Dis. Model Mech.* 12, dmm036186. 10.1242/dmm.036186 30642871 PMC6398497

[DMM049361C14] Mcilroy, G. D., Mitchell, S. E., Han, W., Delibegović, M. and Rochford, J. J. (2020). Ablation of Bscl2/seipin in hepatocytes does not cause metabolic dysfunction in congenital generalised lipodystrophy. *Dis. Model Mech.* 13, dmm042655. 10.1242/dmm.042655 31848133 PMC6994952

[DMM049361C15] O'Brien, P. D., Guo, K., Eid, S. A., Rumora, A. E., Hinder, L. M., Hayes, J. M., Mendelson, F. E., Hur, J. and Feldman, E. L. (2020). Integrated lipidomic and transcriptomic analyses identify altered nerve triglycerides in mouse models of prediabetes and type 2 diabetes. *Dis. Model Mech.* 13, dmm042101. 10.1242/dmm.042101 31822493 PMC6994925

[DMM049361C16] Otto, G. W., Kaisaki, P. J., Brial, F., Le Lay, A., Cazier, J.-B., Mott, R. and Gauguier, D. (2019). Conserved properties of genetic architecture of renal and fat transcriptomes in rat models of insulin resistance. *Dis. Model Mech.* 12, dmm038539. 10.1242/dmm.038539 31213483 PMC6679378

[DMM049361C17] Renner, S., Martins, A. S., Streckel, E., Braun-Reichhart, C., Backman, M., Prehn, C., Klymiuk, N., Bähr, A., Blutke, A., Landbrecht-Schessl, C. et al. (2019). Mild maternal hyperglycemia in INSC93S transgenic pigs causes impaired glucose tolerance and metabolic alterations in neonatal offspring. *Dis. Model Mech.* 12, dmm039156. 10.1242/dmm.039156 31308048 PMC6737953

[DMM049361C18] Resaz, R., Cangelosi, D., Morini, M., Segalerba, D., Mastracci, L., Grillo, F., Bosco, M. C., Bottino, C., Colombo, I. and Eva, A. (2020). Circulating exosomal microRNAs as potential biomarkers of hepatic injury and inflammation in a murine model of glycogen storage disease type 1a. *Dis. Model Mech.* 13, dmm043364. 10.1242/dmm.043364 32620541 PMC7520457

[DMM049361C19] Roostalu, U., Lercke Skytte, J., Gravesen Salinas, C., Klein, T., Vrang, N., Jelsing, J. and Hecksher-Sørensen, J. (2020). 3D quantification of changes in pancreatic islets in mouse models of diabetes type I and II. *Dis. Model Mech.* 13, dmm045351. 10.1242/dmm.045351 33158929 PMC7758639

[DMM049361C20] Sembach, F. E., Ægidius, H. M., Fink, L. N., Secher, T., Aarup, A., Jelsing, J., Vrang, N., Feldt-Rasmussen, B., Rigbolt, K. T. G., Nielsen, J. C. et al. (2021). Integrative transcriptomic profiling of a mouse model of hypertension-accelerated diabetic kidney disease. *Dis. Model Mech.* 14, dmm049086. 10.1242/dmm.049086 34494644 PMC8560499

[DMM049361C21] Seaquist, E. (2021). Celebrating 100 years of insulin with Dr Elizabeth Seaquist. *Dis. Model Mech.* 14, dmm049351. 10.1242/dmm.049351 34762125 PMC8592015

[DMM049361C25] Sims, E. K., Carr, A. L. J., Oram, R. A., DiMeglio, L. A. and Evans-Molina, C. (2021). 100 years of insulin: celebrating the past, present and future of diabetes therapy. *Nat. Med.* 27, 1154-1164. 10.1038/s41591-021-01418-2 34267380 PMC8802620

[DMM049361C26] Shapiro, A. M., Lakey, J. R., Ryan, E. A., Korbutt, G. S., Toth, E., Warnock, G. L., Kneteman, N. M. and Rajotte, R. V. (2000). Islet transplantation in seven patients with type 1 diabetes mellitus using a glucocorticoid-free immunosuppressive regimen. *N. Engl. J. Med.* 343, 230-238. 10.1056/NEJM200007273430401 10911004

[DMM049361C22] Traxler, L., Lagerwall, J., Eichhorner, S., Stefanoni, D., D'Alessandro, A. and Mertens, J. (2021). Metabolism navigates neural cell fate in development, aging and neurodegeneration. *Dis. Model Mech.* 14, dmm048993. 10.1242/dmm.048993 34345916 PMC8353098

[DMM049361C23] Villar-Lorenzo, A., Rada, P., Rey, E., Marañón, P., Arroba, A. I., Santamaría, B., Sáiz, J., Rupérez, F. J., Barbas, C., García-Monzón, C. et al. (2019). Insulin receptor substrate 2 (IRS2) deficiency delays liver fibrosis associated with cholestatic injury. *Dis. Model Mech.* 12, dmm038810. 10.1242/dmm.038810 31262748 PMC6679376

[DMM049361C24] Walls, S. M., Chatfield, D. A., Ocorr, K., Harris, G. L. and Bodmer, R. (2020). Systemic and heart autonomous effects of sphingosine Δ4 desaturase deficiency in lipotoxic cardiac pathophysiology. *Dis. Model Mech.* 13, dmm043083. 10.1242/dmm.043083 32641420 PMC7438009

